# The New Local Anæsthetic—Hydrochlorate of Cocaine

**Published:** 1884-12

**Authors:** 


**THE NEW LOCAL ANÆSTHETIC—HYDROCHLORATE OF COCAINE.**—A New Local Anæsthetic known as hydrochlorate or muriate of cocoaine has been recently discovered and applied first in Germany and later in this country in operations upon the eyes, with very astonishing and satisfactory results. Its farmula is C, 17 H21 N O4, being the alkaloid of the leaves of the *erythroxylon coca*, which have long been used by the natives of Peru and Bolivia as a nerve stimulant. Animals may be destroyed by it, on account of its causing paralysis of the respiratory centres.

Experiments recently made by the writer with this new local anæsthetic, have demonstrated its anæsthetic effect upon tooth-pulp tissue and sensitive dentine. The two and four per cent. solutions were employed, made with distilled water and Merk's crystals of hydrochlorate cocaine applied to the sensitive surface three times at intervals of fifteen minutes. At the expiration of twenty-five and thirty minutes, a very decided anæsthetic effect was produced, and at the expiration of forty minutes pulp tissue was removed without pain, and the sensitiveness of dentine obtunded without pain on its application.

Experiments made with this new agent as a local anæsthetic for the extraction of teeth, were not however, successful, as its anæsthetic effects appear to be manifested only upon such delicate tissues as the conjunctive of the eye, and pulp structure. A stronger solution, if such can be obtained, than the four per cent. solution, may obtund even gum tissue, but all experiments upon such tissue have as yet failed to produce the required anæsthetic effect.

